# Sustained Hippocampal Synaptic Pathophysiology Following Single and Repeated Closed-Head Concussive Impacts

**DOI:** 10.3389/fncel.2021.652721

**Published:** 2021-03-31

**Authors:** John McDaid, Clark A. Briggs, Nikki M. Barrington, Daniel A. Peterson, Dorothy A. Kozlowski, Grace E. Stutzmann

**Affiliations:** ^1^Center for Neurodegenerative Disease and Therapeutics, Rosalind Franklin University of Medicine and Science, North Chicago, IL, United States; ^2^Chicago Medical School, Rosalind Franklin University of Medicine and Science, North Chicago, IL, United States; ^3^Center for Stem Cell and Regenerative Medicine, Rosalind Franklin University of Medicine and Science, North Chicago, IL, United States; ^4^Department of Biological Sciences and Neuroscience Program, DePaul University, Chicago, IL, United States

**Keywords:** Ca^2+^, CTE, concussion, hippocampus, LTP, synaptic transmission, tau

## Abstract

Traumatic brain injury (TBI), and related diseases such as chronic traumatic encephalopathy (CTE) and Alzheimer’s (AD), are of increasing concern in part due to enhanced awareness of their long-term neurological effects on memory and behavior. Repeated concussions, vs. single concussions, have been shown to result in worsened and sustained symptoms including impaired cognition and histopathology. To assess and compare the persistent effects of single or repeated concussive impacts on mediators of memory encoding such as synaptic transmission, plasticity, and cellular Ca^2+^ signaling, a closed-head controlled cortical impact (CCI) approach was used which closely replicates the mode of injury in clinical cases. Adult male rats received a sham procedure, a single impact, or three successive impacts at 48-hour intervals. After 30 days, hippocampal slices were prepared for electrophysiological recordings and 2-photon Ca^2+^ imaging, or fixed and immunostained for pathogenic phospho-tau species. In both concussion groups, hippocampal circuits showed hyper-excitable synaptic responsivity upon Schaffer collateral stimulation compared to sham animals, indicating sustained defects in hippocampal circuitry. This was not accompanied by sustained LTP deficits, but resting Ca^2+^ levels and voltage-gated Ca^2+^ signals were elevated in both concussion groups, while ryanodine receptor-evoked Ca^2+^ responses decreased with repeat concussions. Furthermore, pathogenic phospho-tau staining was progressively elevated in both concussion groups, with spreading beyond the hemisphere of injury, consistent with CTE. Thus, single and repeated concussions lead to a persistent upregulation of excitatory hippocampal synapses, possibly through changes in postsynaptic Ca^2+^ signaling/regulation, which may contribute to histopathology and detrimental long-term cognitive symptoms.

## Introduction

Traumatic brain injury (TBI) is a leading cause of death and disability in the US, affecting approximately 2.8 million people annually, resulting in a significant financial and human toll (Taylor et al., 2017). Numerous postmortem studies reveal a correlation between repeated concussion and chronic traumatic encephalopathy (CTE), a neurodegenerative condition characterized by cognitive impairment, mood swings, and depression, and disrupts vulnerable brain regions involved in memory processing such as the cortex and hippocampus (McKee et al., 2009, 2013; McKee and Robinson, 2014). While many high profile reports of TBI are associated with military and athletic events, a greater proportion of the general public is affected by concussions resulting primarily from falls and violence (CDC, 2014), with single or repeated concussions resulting in short-term memory loss (Mayer et al., 2017) or other symptoms of neurodegenerative disease (Fakhran et al., 2013). Indeed, TBI-related CTE bears many of the same pathophysiological hallmarks as Alzheimer’s disease, including the buildup of hyperphosphorylated tau, a microtubule-associated protein involved in cytoskeletal function (Geddes et al., 1999; McKee and Robinson, 2014). Long-term effects of TBI also include increased likelihood of seizures (Annegers et al., 1998) and loss of balance/abnormal gait (Ellemberg et al., 2009), all of which further increase the likelihood of subsequent TBIs (Theadom et al., 2015). Although the long-term underlying physiology has not been well studied, the acute effects of a concussion are thought to result from several abnormal cellular processes occurring in the wake of a concussive impact. Upon impact, there is a rapid increase in extracellular glutamate (Hinzman et al., 2010), resulting in over-activation of postsynaptic Ca^2+^ channels including NMDA receptors (NMDARs; Geddes et al., 2003; Biegon et al., 2004) and voltage gated Ca^2+^ channels (VGCCs; Wolf et al., 2001). The consequences of increased neuronal Ca^2+^ may persist for hours or even days after impact (Sun et al., 2008) and are often accompanied by some form of cognitive impairment (Deshpande et al., 2008). The sustained alteration of Ca^2+^ homeostasis is a central feature of several neurological diseases including Alzheimer’s and Huntington’s diseases and may serve as a common underlying mechanism linking increased incidence of dementia in the years after a TBI (Giacomello et al., 2013; Schrank et al., 2020).

Ca^2+^ is a vitally important mediator of normal cellular and synaptic function. Neuronal Ca^2+^ is tightly regulated through several homeostatic mechanisms with disruption of these processes contributing to synaptic dysregulation, altered signaling cascades, and potential cell injury or death (Stutzmann, 2007; Chakroborty et al., 2009, 2019; Zhang et al., 2015). Postsynaptic ryanodine receptors (RyRs), which are high conductance Ca^2+^ channels on the endoplasmic reticulum (ER) membrane, are located close to NMDARs and VGCCs on dendritic spines of hippocampal pyramidal cells (Jaffe et al., 1994; Chavis et al., 1996; Thibault et al., 2007; Holbro et al., 2009). This relationship allows for the amplification of NMDAR and VGCC mediated Ca^2+^ influx through the process of Ca^2+^ induced Ca^2+^ release (CICR; Emptage et al., 1999; Borde et al., 2000; Holbro et al., 2009), and as such, RyRs are positioned to influence Ca^2+^-dependent synaptic and plasticity processes (Chakroborty et al., 2009, 2019). Also, RyR expression is elevated in postmortem brain tissue from individuals with cognitive impairment, a feature of TBI (Bruno et al., 2012; McInnes et al., 2019). RyR sensitivity is altered by the processes of phosphorylation and oxidation which increase channel open probability, resulting in spontaneously “leaky” channels and increased Ca^2+^ release in the short term, but reduced ER store content and suppressed ER Ca^2+^ release if sustained (Liu et al., 2012; Lacampagne et al., 2017). Although the role of RyRs in the short-term effects of TBI is not well understood, TBI acutely results in an increased production of reactive oxygen species (ROS) which are known mediators of RyR hyperphosphorylation/oxidation (Görlach et al., 2015). As RyRs are also expressed presynaptically, increased presynaptic RyR sensitivity/Ca^2+^ may result in increased release of the excitatory neurotransmitter glutamate, resulting in feed-forward increased postsynaptic NMDAR and RyR-mediated Ca^2+^ release. Notably, stabilization of RyR-evoked Ca^2+^ release results in wide-ranging therapeutic effects, including reduced glutamate excitotoxicity (Frandsen and Schousboe, 1991; Niebauer and Gruenthal, 1999), normalized synaptic transmission and synaptic structure, reduced inflammatory markers, and improved behavioral outcomes (Chakroborty et al., 2012; Oules et al., 2012; Briggs et al., 2017). Thus, RyRs may emerge as a therapeutic target in the treatment of TBI and related disease conditions.

TBI generates several pathological features common to other neurological diseases, including abnormal protein aggregates and hyperphosphorylated tau. Under normal conditions, homeostasis of tau phosphorylation is maintained through a balance of kinase and phosphatase activity responsible for its phosphorylation and dephosphorylation, respectively (Geddes et al., 1999; McKee and Robinson, 2014). After TBI, multiple kinases are upregulated and result in a net increase of phosphorylated tau (Tran et al., 2012). Furthermore, increased neuronal Ca^2+^ following TBI, *via* NMDARs, VGCCs, and intracellular stores can upregulate specific Ca^2+^-regulated kinases that phosphorylate tau, such as GSK3-β and Cdk5 (Avila et al., 2004; Dash et al., 2011; Zhao et al., 2012; Wilson et al., 2014). In turn, phosphorylated tau can increase intracellular Ca^2+^, furthering tau phosphorylation (Gómez-Ramos et al., 2006; Stutzmann, 2007) and Ca^2+^-related synaptic deficits. While acute excitotoxic Ca^2+^ events have been described in the minutes to hours following a TBI (Luo et al., 2011; Gurkoff et al., 2013; Arai et al., 2019), sustained intracellular Ca^2+^ dyshomeostasis, such as that seen in AD (Stutzmann, 2007), may also occur and underlie cognitive, histopathological, and synaptic defects that can arise weeks to months after injury (Deshpande et al., 2008; Sun et al., 2008).

Previous head injury is a significant risk factor for dementia-related diseases, with the delay from injury to onset of dementia-like symptoms ranging from months to years (Fleminger et al., 2003; Li et al., 2017). RyRs and VGCCs each play an important role in Ca^2+^ homeostasis, synaptic transmission, and memory encoding, and despite the documented role of Ca^2+^ dysregulation in neurodegenerative diseases (Huang and Malenka, 1993; Huber et al., 1995; Chakroborty et al., 2012; Oules et al., 2012), their contribution to the sustained cellular and synaptic defects resulting from TBI has not been adequately studied. Here we investigate modes of sustained pathophysiology resulting from single or repeated TBI in a clinically-relevant rat model (Jamnia et al., 2017), and reveal key cellular signaling, synaptic circuit defects, and histopathological markers that are consistent with chronic neurological disease states.

## Materials and Methods

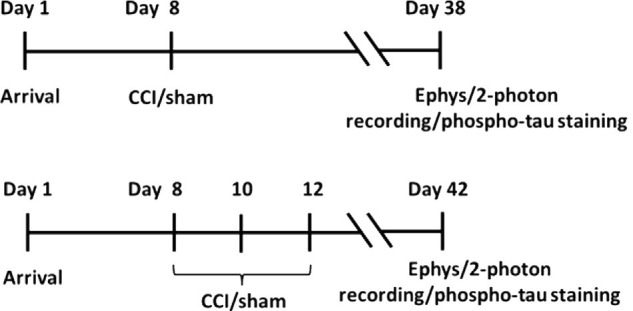



### Timeline of the Experimental Procedure

Approximately 1 week after arrival, animals were subjected to sham surgery, or single or repeated closed-head controlled cortical impacts (CCI). Repeated CCI’s were conducted using three successive impacts separated by 48-h intervals. Rats were examined 30 days after the last CCI to measure the extent of sustained synaptic and cellular effects; see the depiction below. Electrophysiology/2-photon recordings and phospho-tau staining were conducted using separate sets of animals.

### Animals

Male hooded Long-Evans rats (Charles River Laboratory; 200– 300g; P60-P80) were housed two per cage in the Rosalind Franklin University of Medicine and Science (RFUMS) Biological Resource Facility. While we acknowledge the importance of sex as a biological variable, the limited scale of the study, along with the much higher incidence of TBI in males (CDC, 2014) means we used only male rats in this study. Rats were kept on a 12:12 h light/dark cycle with food and water available *ad libitum*. Animals were handled daily for approximately 1 week before surgery. All experiments were performed following the National Institutes of Health Guide for the Care and Use of Animals and were approved by the RFUMS Institutional Animal Care and Use Committee.

### Closed-Head Controlled Cortical Impact (CCI)

To inflict a closed-head TBI we used a modified CCI approach (Leica Impact One, Leica Microsystems Inc., Buffalo Grove, IL, USA) to model mild TBI (Jamnia et al., 2017). Rats were anesthetized using 2.0–3.0 ml/min isoflurane anesthesia and placed in a Kopf stereotaxic apparatus (Kopf, Tujunga, CA, USA), on a foam bed (5 cm thick) in a plexiglass frame; anesthesia was maintained through a nose cone. The frame consisted of a base (11.43 × 24.13 cm) and a side piece (9.52 × 22.86 cm) angled 11 degrees from the vertical. To allow for movement of the head, but to also provide stability to the rat during the impact, the lateral surface of the head was rested lightly against the plexiglass frame. The Impact One stereotactic device delivered the cortical impact at an angle 20 degrees from vertical, enabling the flat impactor tip to be perpendicular to the surface of the head; this was adjusted accordingly if necessary. All injuries were produced with a 5 mm flat tip at 6.5 m/s at a depth of 10 mm from the surface of the skin overlaying the right sensorimotor cortex for 300 ms. The depth measurement accounted for the amount of absorption in the foam (approximately 9 mm). This depth was previously tested to be the maximal level of impact that did not result in a skull fracture but produced behavioral impairment (Jamnia et al., 2017). All rats that received an impact received a topical analgesic, and body temperature was maintained at 37°C during recovery. Following recovery from anesthesia, rats were returned to their home cages and monitored daily. Repeated concussion animals had a total of three injuries 48 h apart; an additional group of rats with a single concussion were exposed to two subsequent anesthesia treatments 48 h apart. This group was placed in the anesthesia chamber for the duration of time that the repeated concussion animals were anesthetized for their subsequent injuries (approximately 20 min). This model of single or repeated TBI, previously used by us, does not result in any obvious tissue damage (Jamnia et al., 2017), and is considered a model of single or repeated mild TBI or concussion (Petraglia et al., 2014).

### Brain Slice Preparation

Thirty days after the last CCI, animals were deeply anesthetized with isoflurane and coronal hippocampal slices (400 μm for field recording and 250 μm for whole-cell recording and Ca^2+^ imaging) were prepared as previously described (Stutzmann et al., 2004). Slices were perfused at 1.5–2 ml/min with standard artificial cerebrospinal fluid (aCSF) solution containing the following (in mM): 125 NaCl, 2.5 KCl, 2 CaCl_2_, 1.2 MgSO_4_, 1.25 NaH_2_PO_4_, 25.0 NaHCO_3_, 10 D-dextrose equilibrated with 95% O_2_ and 5% CO_2_ (pH 7.3–7.4), at room temperature (22°C). Osmolarity was maintained at 310 mOsm. Patch pipettes (4–5 MΩ) were filled with intracellular solution containing the following substances (in mM): 135 K-gluconate, 10 HEPES, 10 Na-phosphocreatine, 2 MgCl_2_, 4 NaATP, and 0.4 NaGTP, pH adjusted to 7.3–7.4 with KOH (Sigma). Hippocampal CA1 pyramidal neurons were identified visually *via* infrared differential interference contrast optics (IRDIC) on an Olympus BX51 upright microscope, through a 40× objective, and were identified electrophysiologically by their passive membrane properties and spike frequency adaptation in response to depolarizing current injection. Membrane potentials were obtained in current-clamp mode acquired at 10 kHz with a Digidata 1322 A-D converter and Multiclamp 700B amplifier and were recorded and analyzed using pClamp 10.2 software (Molecular Devices).

### Extracellular Field Potential Recordings

**Figure 1:**
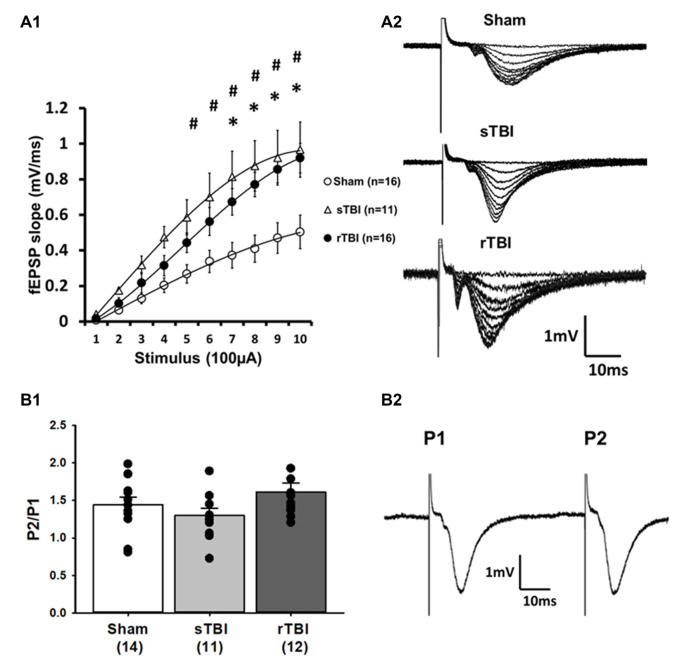
Single or repeated traumatic brain injury (TBI) results in persistent hyperexcitability at CA3-CA1 synapses. Rats subjected to a single TBI (sTBI), or repeated TBI (rTBI), exhibited persistent upregulation at CA3-CA1 synapses, as revealed using extracellular field recordings (fEPSPs). **(A1)** fEPSP Input-output curves obtained from CA1 hippocampal field recordings using Schaffer collateral stimulation, 30 days after sham surgery, sTBI or rTBI, demonstrating increasing field potential (fEPSP) slopes resulting from increasing stimulus intensity ranging from 100 μA to 1,000 μA. fEPSPs from both the sTBI and rTBI groups showed a steeper slope at most stimulus intensities compared to the sham treatment group, with no significant difference between the sTBI and rTBI groups. **(A2)** Representative input-output fEPSP traces taken from sham, sTBI, and rTBI rats. **(B1)** sTBI and rTBI did not affect the paired-pulse ratio of fEPSPs, compared to sham, indicating that there was no significant difference in the presynaptic release probability of glutamate between groups. **(B2)** Representative traces showing paired fEPSPs evoked at a 50 ms interstimulus interval. All recordings were on the hippocampal side ipsilateral to the TBI impact site (over the right sensorimotor cortex). ^#^*p* < 0.05 for sham vs. sTBI. *p < 0.05.for sham vs. rTBI. Error bars represent the standard error of the mean.

For extracellular field potential recording, 400 μm hippocampal slices were transferred to an interface chamber (Harvard Apparatus), perfused with oxygenated aCSF (1.5 ml/min) at room temperature, and covered with a continuous flow of humidified gas (95% O_2_/5% CO_2_). Data were acquired at 10 kHz using pClamp 10.2 software with an AxoClamp 2B amplifier and a DigiData 1322A board for digitization (Molecular Devices). Field excitatory postsynaptic potentials (fEPSPs) were recorded in the stratum radiatum of the CA1 subfield of the hippocampus using recording microelectrodes (2–6 MΩ) filled with aCSF. Microelectrodes were pulled from borosilicate glass capillaries (Harvard Apparatus) on a P-2000 pipette puller (Sutter Instruments, Novato, CA, USA). Synaptic fEPSP responses were evoked by stimulation of the Schaffer collateral/commissural pathway, using a bipolar stimulating electrode, with the fEPSP slope calculated as the change in potential (ΔV) of the initial fEPSP waveform over time (t), or ΔV/t (mV/ms; see Figure 1A). Input-stimulus/output-fEPSP slope (I-O) curves were generated by plotting the fEPSP slope vs. stimulus intensity, using stimulus intensities ranging from 100 to 1,000 μA. For induction of long-term potentiation (LTP), baseline fEPSPs were evoked at 50% of the maximum fEPSP slope, at 0.05 Hz for 20 min. LTP was induced at baseline intensity using a high-frequency stimulation (HFS) protocol consisting of two sets of 100 pulses at 100 Hz, with each set of pulses spaced 10 sapart. fEPSPs were recorded for a further 60 min at baseline frequency, after HFS. Immediately after HFS, we observed post-tetanic potentiation (PTP; 0–2 min after HFS), a Ca^2+^ dependent form of short-term synaptic plasticity (Zucker and Regehr, 2002). LTP was observed later and can be divided into a transient early phase that is mediated by modification of pre-existing proteins (E-LTP; 15–20 min after HFS), and a more persistent late phase that requires gene transcription and new protein synthesis (L-LTP; 50–60 min after HFS; Davies et al., 1989; Frey et al., 1993). Paired-pulse facilitation (PPF) was assessed after I-O recordings, using an interstimulus interval of 50 ms. Three successive response pairs were recorded at 0.05 Hz. All field recordings were performed on the hippocampal side ipsilateral to the impact site. Only one set of field recordings was carried out per slice, with typically two 400 μm hippocampal slices per rat used.

### Ca^2+^ Imaging

Ca^2+^ imaging within individual CA1 pyramidal neurons was performed in hippocampal slices using a custom-built video-rate multiphoton-imaging system based on an upright Olympus BX51 microscope frame and Ti:Saph pulsed IR laser (Spectra-Physics, Santa Clara, CA, USA; Stutzmann and Parker, 2005). Individual neurons were filled with the Ca^2+^ indicator fura-2 (50 μM; ThermoFisher) *via* the patch pipette as described previously (Stutzmann et al., 2004). Laser excitation was provided by trains (80 MHz) of ~100 frames/s pulses at 780 nm. At this wavelength, fura-2 excitation generates a bright signal at low Ca^2+^ levels, allowing us to see small compartments such as spines at resting levels; fluorescence proportionally decreases with increasing Ca^2+^ levels allowing for relative measurements of change from baseline. The femtosecond pulsed laser beam was scanned by a resonant galvanometer (General Scanning Lumonics), allowing rapid (7.9 kHz) bidirectional scanning in the *x*-axis and by a conventional linear galvanometer in the *y*-axis, to provide a full-frame scan rate of 30 frames/sec. The laser beam was focused onto the tissue through a 40× water-immersion objective (numerical aperture = 0.8). Emitted fluorescence light was detected by a wide-field photomultiplier (Electron Tubes) to derive a video signal that was captured and analyzed by Video Savant 5.0 software (IO Industries; London, ON). Further analysis of background-corrected images was performed off-line using MetaMorph software. For quantification of resting and changes in fura-2 fluorescence, we selected a region of the CA1 pyramidal cell soma (surrounding but excluding the nucleus), to obtain a measure of cytosolic Ca^2+^ (Stutzmann et al., 2003, 2004). An adjacent non-fluorescent area was selected for background subtraction. As fura-2 shows a decrease in fluorescence with increasing Ca^2+^, using two-photon excitation at 780 nm, decreased basal *F*_0_values represent increased basal Ca^2+^. Relative percentage changes in Ca^2+^ were calculated as the percentage change in fluorescence intensity over baseline: (*F*_0_/Δ*F* − 1) × 100 with values for *F*_0_ and percentage change averaged across cells from each TBI group. After patching, fura-2 was allowed to equilibrate in the cell for 10–15 min, and after a fluorescent signal was attained, the positive current was injected into the cell while in current-clamp mode, resulting in depolarization of the cell membrane and triggering activation of voltage-gated Ca^2+^ channels (VGCCs). To ensure consistency between evoked responses within and between groups, the depolarization protocol was conducted first, before any drug exposure. Ca^2+^ measurements were acquired from the peak of the evoked response. Bath application of caffeine (10 mM) was used to trigger RyR-Ca^2+^ release from ER stores (Garaschuk et al., 1997; Sandler and Barbara, 1999). Each brain slice was exposed to only one application of caffeine. Typically on any recording day, two hippocampal slices were used for Ca^2+^ measurements with one CA1 pyramidal cell recording per slice. Minianalysis 6.0.7 (Synaptosoft; Fort Lee, NJ, USA) was used to detect and measure spontaneous excitatory postsynaptic potential (sEPSP) events with a minimal amplitude of 0.2 mV and minimal area of 3 mV×ms. The baseline was determined from a 1 ms average immediately before each event, or in the case of overlapping events, from the peak and decay kinetics of the preceding event using “complex peak detection” in Minianalysis. 6-Cyano-7-nitroquinoxaline-2, 3-dione (CNQX, Sigma-Aldrich), an AMPA antagonist, was used to confirm glutamatergic sEPSPs. sEPSP data was obtained in the sham and rTBI groups only, as we only observed effects of rTBI, but not sTBI, on the somatic RyR-Ca^2+^ response to caffeine (see Figure 4). All whole-cell/2-photon recordings were performed on the hippocampal side ipsilateral to the impact site. Multiple VGCC recordings were carried out per slice, but only one RyR-Ca^2+^ response to caffeine per slice, with typically 2–3 250 μm hippocampal slices per rat used.

**Figure 2:**
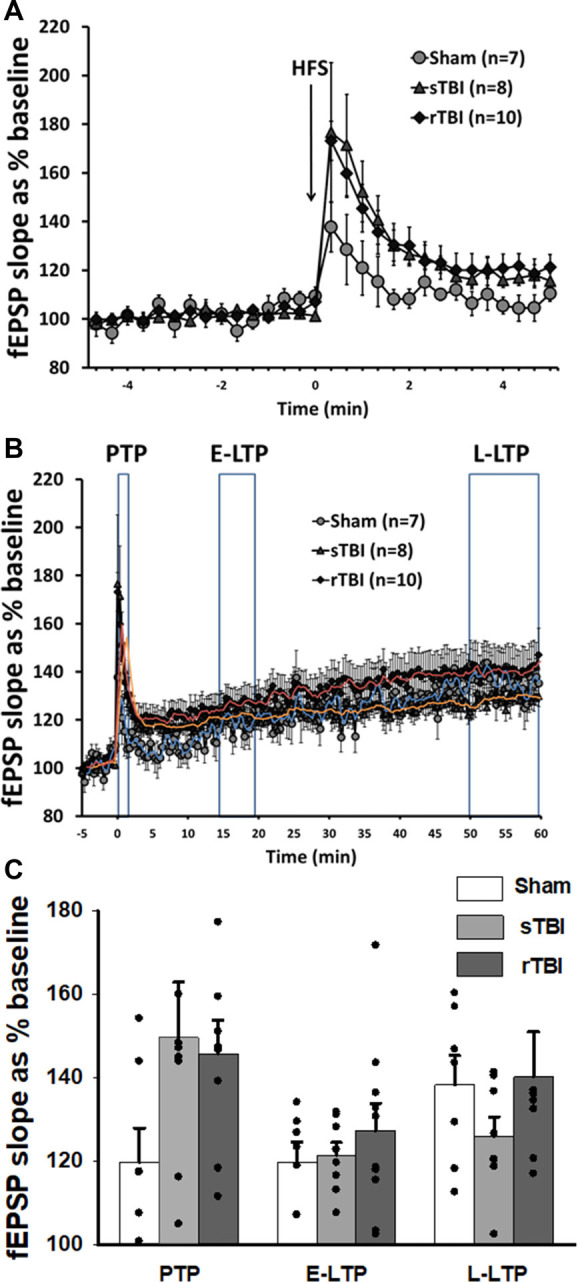
Acute induction of hippocampal synaptic plasticity is unaffected by single or repeated TBI. **(A)** High-frequency stimulation (HFS) of CA3-CA1 Schaffer collaterals resulted in short-term post-tetanic synaptic potentiation (PTP), lasting for several minutes. Graph shows averaged time course of PTP in sham, single TBI (sTBI), and repeated TBI (rTBI) groups. **(B)** High-frequency stimulation (HFS) of CA3-CA1 Schaffer collaterals also resulted in long-term potentiation (LTP) lasting for 1 h. Graph shows averaged time course of early (E-LTP) and late-phase LTP (L-LTP) in sham, sTBI, and rTBI groups. Time of HFS indicated by the arrow. **(C)** Bar graph shows the percentage change in post-HFS response relative to baseline as PTP (0–2 min after HFS), E-LTP (15–20 min after HFS), and L-LTP (50–60 min after HFS). HFS consisted of two 100 Hz pulse trains of 1 s duration each, with a 10 s interval between pulse trains. There was no significant effect of sTBI or rTBI on the magnitude of PTP, E-LTP, or L-LTP. Superimposed trendlines represent mean data for sham (blue), sTBI (orange), and rTBI (red). All recordings were on the hippocampal side ipsilateral to the TBI impact site (over the right sensorimotor cortex). Error bars represent the standard error of the mean.

**Figure 3:**
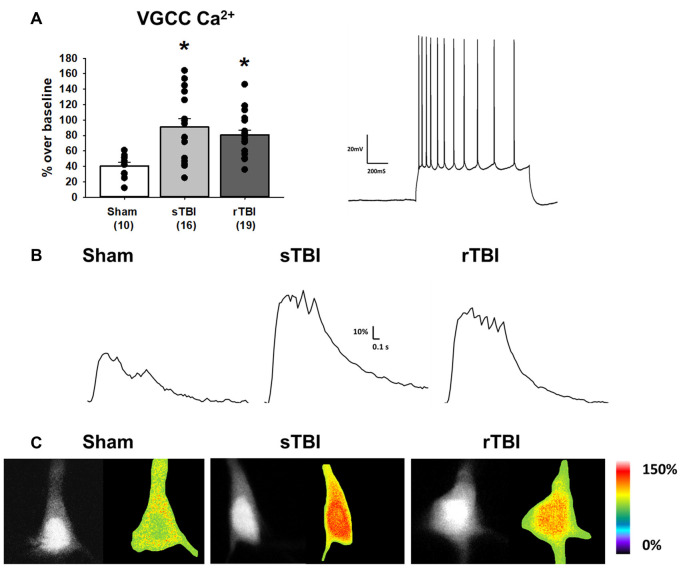
TBI results in enhanced voltage-gated Ca^2+^ channel activity in hippocampal CA1 pyramidal neurons. **(A)** Left, bar graph summarizing peak Ca^2+^ effects of voltage-gated Ca^2+^ channel (VGCC) activation (using a depolarizing current injection in CA1 pyramidal neurons) 30 days after either a sham procedure, single TBI (sTBI), or repeated TBI (rTBI). Peak VGCC Ca^2+^ effects were larger in both the sTBI (gray bar) and rTBI (black bar) groups relative to sham (white bar). The number of recorded cells per group is in each bar. Right, action potential train in response to a depolarizing current injection in a CA1 pyramidal neuron (action potential characteristics were unchanged by either sTBI or rTBI, see Table 1). **(B)** Sample traces illustrating Ca^2+^ effects of VGCC activation in individual sham (left), sTBI (middle) or rTBI (right) CA1 pyramidal neurons. Calibration bars illustrate the % Ca^2+^ change over baseline. **(C)** Fluorescent (monochrome) and pseudocolored images of fura-2 loaded CA1 pyramidal neurons. Pseudocolored images illustrate relative Ca^2+^ responses to VGCC activation, from sham (left), sTBI (middle) and rTBI (right) groups. *p < 0.05. All recordings were on the hippocampal side ipsilateral to the TBI impact site (over the right sensorimotor cortex). Error bars represent the standard error of the mean.

**Figure 4:**
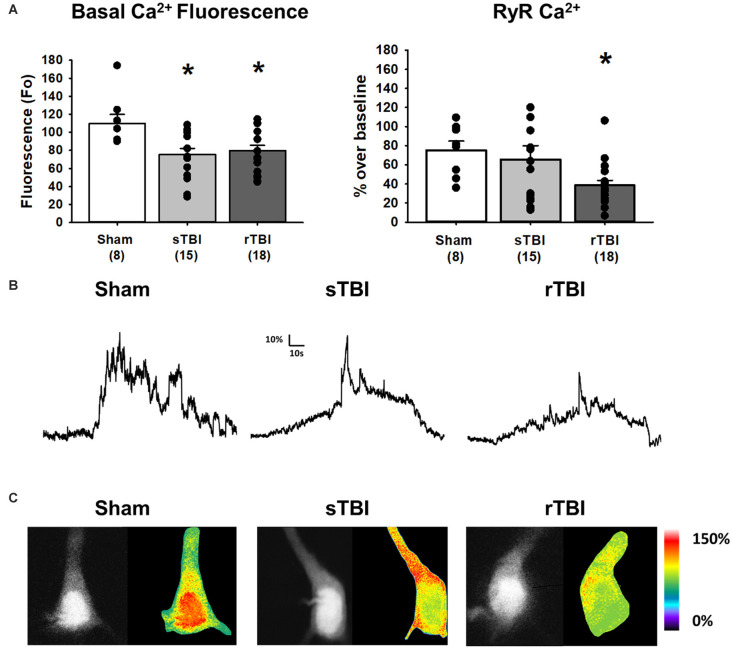
TBI results in increased resting Ca^2+^ and blunted RyR-evoked Ca^2+^ release in hippocampal CA1 pyramidal neurons. **(A)** Left, bar graph summarizing averaged resting Ca^2+^-based fluorescence (*F*_0_) in CA1 pyramidal neurons 30 days after either a sham procedure (white bar), single TBI (sTBI, gray bar), or repeated TBI (rTBI, black bar). The decreased resting fluorescence (*F*_0_) values of both TBI groups compared to the sham group are indicative of increased resting Ca^2+^. Right, a bar graph illustrating that the averaged RyR-Ca^2+^ response to caffeine (20 mM) was decreased in the rTBI group (black bar) but not the sTBI group (gray bar), relative to sham (white bar). The number of recorded cells per group is in each bar. **(B)** Sample traces illustrating RyR-Ca^2+^ effects of caffeine (20 mM), in individual sham (left), sTBI (middle), and rTBI (right) CA1 pyramidal neurons. Calibration bars illustrate the % Ca^2+^ change over baseline. **(C)** Fluorescent (monochrome) and pseudocolored images of fura-2 loaded CA1 pyramidal neurons. Pseudocolored images illustrate relative RyR-Ca^2+^ effects of caffeine, from sham (left), sTBI (middle), and rTBI (right) groups. *p < 0.05. All recordings were on the hippocampal side ipsilateral to the TBI impact site (over the right sensorimotor cortex). Error bars represent the standard error of the mean.

### Immunohistochemistry

Rats were anesthetized with pentobarbital (200 mg/kg) and then transcardially perfused with ice-cold phosphate-buffered saline, immediately followed by 4% paraformaldehyde. Brains were removed and post-fixed for 24 h, then cut into sagittal sections on a Leica SM2000R microtome with a freezing stage at 40 μm thickness and stored in cryopreservation solution until immunostaining. Free-floating hippocampal sections were permeabilized with 0.1 M TBS 1.0% Triton-X (3 × 10 min) at room temperature on a platform rocker. Sections were blocked with 0.1 M TBS 0.1% Triton-X + 10% goat serum for 1 h at room temperature and subsequently incubated in primary antibody (phospho-Tau S262, 1:500, Invitrogen, catalog number 44-750G) diluted in 0.1 M TBS 0.1% Triton-X + 1% goat serum for 24 h at 4°C on a platform rocker. The sections were washed with 0.1 M TBS 0.1% Triton-X (3 × 5 min) and incubated in secondary antibody (Alexa Fluor 488 conjugated to IgG goat anti-rabbit antibody, 1:1,000, Invitrogen, A-11008) diluted in 0.1 M TBS 0.1% Triton-X + 1% goat serum for one hour at room temperature on a platform rocker. Sections were rinsed in 0.1 M TBS 0.1% Triton-X (3 × 5 min) and stained with 1:10,000 DAPI diluted in 0.1 M TBS for 5 min. Sections were washed in 0.1 M TBS for 5 min and mounted with PVA-DABCO for microscopy. Control sections were obtained using the same procedure with primary antibody alone and secondary antibody alone, respectively, and imaged to ensure there was no off-target staining. Confocal images of the sections were obtained and analyzed using a 60× objective lens on an Olympus Fluoview Fv10i confocal microscope. Microscope settings were the same for all images. Several images were obtained of each hippocampal region as well as the cortex. MetaMorph software (Version 7) was used to quantify the percent staining density of fluorescently labeled phosphorylated tau over the threshold intensity level across the whole brain as well as in each hemisphere ipsilateral and contralateral to injury.

### Statistical Analysis

Statistical differences among groups were determined using analysis of variance (one-way ANOVA or two-way repeated-measures ANOVA) with Tukey or Dunnett’s *post hoc* tests where appropriate using SigmaPlot 12 data analysis software. Data are shown as mean ± standard error of the mean. Statistical significance was set at *p*<0.05.

## Results

In our previous study, repeated concussion/TBI resulted in more persistent memory deficits than a single concussion (Jamnia et al., 2017), with similar findings observed elsewhere (Meehan et al., 2012; Weil et al., 2014). Concussion-related memory deficits may result from persistent maladaptive pathophysiological effects in brain regions that play a role in learning and memory, such as the hippocampus. To this end, we tested for persistent hippocampal synaptic and neuronal Ca^2+^ signaling deficits, and tau pathology, in rats subjected to single or repeated TBI.

### sTBI and rTBI Results in Hyperexcitable Synaptic Transmission in Hippocampal Circuits

Electrophysiological recordings in acute hippocampal brain slices were used to assess alterations in the local circuit and synaptic function resulting from either a single or repeated TBI. A total of 7, 6, and 8 rats were allocated to the sham, sTBI, and rTBI groups, respectively. Extracellular field potential recordings measured effects of Schaffer collateral CA3-CA1 stimulation on postsynaptic potentials (fEPSPs), using the initial slope function of the fEPSP as a measure of monosynaptic output. Assessment of the input-output function of fEPSPs revealed that both sTBI (11 recordings/11 slices/6 rats) and rTBI (18 recordings/18 slices/8 rats) resulted in significant potentiation at hippocampal synapses, across a range of stimulation intensities, when compared to the sham group (18 recordings/18 slices/8 rats). A two-way repeated-measures ANOVA was significant for effects of single and repeated TBI treatment (*F*_(2,40)_ = 4.08; *p* = 0.024), stimulation intensity (*F*_(9,40)_ = 89.08; *p* < 0.001) and treatment group vs. stimulation-intensity interaction (*F*_(2,9)_ = 4.27; *p* < 0.001). *Post hoc* analysis revealed a significant difference between the sTBI and sham groups at 500 μA (*p* = 0.049), 600 μA (*p* = 0.02), 700 μA (*p* = 0.004), 800 μA (*p* = 0.002), 900 μA (*p* = 0.004), and 1,000 μA (*p* = 0.003) stimulation intensities and between the rTBI and sham groups at 700 μA (*p* = 0.025), 800 μA (*p* < 0.005), 900 μA (*p* < 0.004), and 1,000 μA (*p* < 0.002) stimulation intensities (Figure 1A). To determine if the increased synaptic responses observed in the sTBI and rTBI groups reflect presynaptic alterations, we used the paired-pulse ratio, measured as the ratio of the slope of the second fEPSP to the slope of the first fEPSP of a pair of fEPSPs obtained at a 50 ms interval, as a relative measure of Ca^2+^-dependent neurotransmitter release probability. A one-way ANOVA revealed no effect of either sTBI (11 recordings/11 slices/6 rats) or rTBI (12 recordings/12 slices/8 rats) on the paired-pulse ratio (*F*_(2,34)_ = 1.44; *p* = 0.252; Figure 1B), compared to sham (14 recordings/14 slices/8 rats), indicating that the probability of presynaptically-regulated vesicle release is not significantly different among these groups.

In addition to the measurement of basal synaptic transmission, we also examined long-term synaptic potentiation (LTP) at CA3-CA1 synapses. Using high-frequency stimulation (HFS, two 100 Hz pulse trains for 1 s each, spaced 10 s apart) of Schaffer collaterals, we observed a sequence of synaptic changes consisting of an initial short-term post-tetanic synaptic potentiation (PTP; 0–2 min after HFS; Figure 2A), followed by early LTP (E-LTP; 15–20 min after HFS) and late-phase LTP (L-LTP; 50–60 min after HFS; Figure 2B). PTP was not significantly altered by either single (sTBI; 8 recordings/8 slices/6 rats) or repeated TBI (rTBI; 10 recordings/10 slices/8 rats; one way ANOVA; *F*_(2,24)_ = 2.34; *p* = 0.12), compared to sham (7 recordings/7 slices/7 rats) and there was likewise no effect of sTBI or rTBI on the magnitude of E-LTP (one way ANOVA; *F*_(2,24)_ = 0.57; *p* = 0.57) or L-LTP (one way ANOVA; *F*_(2,24)_ = 0.89; *p* = 0.43; Figure 2C). Although our findings contrast with those from other studies showing a persistent attenuation of hippocampal LTP resulting from sTBI (Schwarzbach et al., 2006) or rTBI (Aungst et al., 2014), these studies used an open skull/exposed dura impact model, and not the closed-head model employed here. Indeed, while many studies have looked at hippocampal synaptic effects resulting from TBI using an open-head/exposed dura model at various time points post-injury (Miyazaki et al., 1992; D’Ambrosio et al., 1998; Atkins, 2011; Zhang B. et al., 2011; Zhang B.-L. et al., 2011; Titus et al., 2019), few studies have examined the persistent TBI effects on synaptic plasticity using a closed-head model of single (White et al., 2017) or repeated impacts (Logue et al., 2016). Also, other closed-head studies have employed a blast injury model (Beamer et al., 2016; Hernandez et al., 2018), with global physical effects that are less clinically relevant to mild TBI.

### Ca^2+^ Dynamics and Membrane Properties in sTBI and rTBI

Neuronal Ca^2+^ dyshomeostasis due to changes in VGCC and RyR function can result in altered cellular signaling and synaptic function, as well as drive synaptic loss and neurodegeneration if sustained over time. Here we have assessed the persistent effects of single or repeated TBI on levels of resting Ca^2+^, VGCC, and RyR function. The use of whole-cell patch recordings with 2-photon Ca^2+^ imaging allows for the simultaneous measurement of passive and active membrane properties, including input resistance and cell excitability, along with changes in Ca^2+^ handling during VGCC and RyR activation. Ca^2+^ influx through VGCCs was significantly increased in both the sTBI (16 recordings/12 slices/6 rats) and rTBI (19 recordings/12 slices/8 rats) groups compared to sham (10 recordings/6 slices/8 rats; *F*_(2,44)_ = 7.89; *p* = 0.001; Figure 3A). Basal Ca^2+^, measured using the resting fura-2 fluorescence intensity (*F*_0_) under identical laser power and recording conditions, was also significantly increased in the both the sTBI (15 recordings/15 slices/6 rats) and rTBI (18 recordings/18 slices/8 rats) groups relative to sham (8 recordings/8 slices/8 rats; *F*_(2,40)_ = 5.0; *p* = 0.012; Figure 4A). In contrast, RyR-evoked Ca^2+^ signals were significantly decreased in the rTBI group (18 recordings/18 slices/8 rats) only (*F*_(2,39)_ = 4.57; *p* = 0.017; Figure 4A), but not sTBI (15 recordings/15 slices/6 rats), compared to sham (8 recordings/8 slices/8 rats). Our findings are consistent with previous studies demonstrating sustained pathophysiological effects resulting from TBI, including increased cortical excitatory synaptic transmission (3–5 weeks post-injury; Koenig et al., 2019) and Ca^2+^ dyshomeostasis in hippocampal neurons at 30 days post-injury (Sun et al., 2008). Also, in both of these studies, the nature of the impact (open-head/exposed dura) was markedly different from the closed-head model used in our study, utilized only a single impact, and used Ca^2+^ imaging data obtained from acutely isolated CA3 hippocampal neurons and not in CA1 pyramidal neurons in a brain slice. Thus, although it is difficult to extrapolate directly across these studies, there is a clear precedent for synaptic defects and Ca^2+^ dyshomeostasis resulting from TBI in brain circuits critical for learning, memory, and cognition.

**Figure 5:**
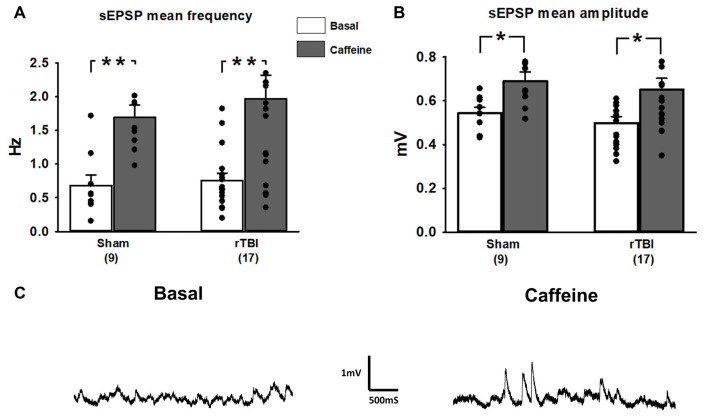
Spontaneous synaptic events and contribution of RyR activation are unaffected by repeated TBI (rTBI). **(A)** The frequency of baseline spontaneous excitatory postsynaptic potentials (sEPSPs; gray bars) measured in hippocampal CA1 pyramidal neurons was not affected by rTBI at 30 days post-injury. sTBI neurons were not tested. In both the sham and rTBI groups, RyR activation by caffeine (20 mM; black bars) increased the frequency of sEPSPs similarly. **(B)** Baseline sEPSP amplitude (gray bars) was also unaffected by rTBI, but RyR activation by caffeine (20 mM; black bars) resulted in a significant increase in sEPSP amplitude in the rTBI group. **(C)** Representative traces illustrating effects of bath application of caffeine (20 mM) on the frequency and amplitude of sEPSPs during a whole-cell current-clamp recording in a CA1 pyramidal neuron from the rTBI group. **p < 0.001, *p < 0.05. All recordings were on the hippocampal side ipsilateral to the TBI impact site (over the right sensorimotor cortex). Error bars represent the standard error of the mean.

In addition to measurement of somatic RyR-evoked Ca^2+^ release, we also examined basal spontaneous synaptic transmission in the form of excitatory postsynaptic potentials (sEPSPs) and how RyR activation differentially affects sEPSC frequency and amplitude in the sham and rTBI groups. As the RyR-Ca^2+^ response was blunted in only the rTBI condition, we similarly tested for sEPSP effects of caffeine in the sham (9 recordings/9 slices/8 rats) and rTBI (17 recordings/17 slices/8 rats) groups. Prior to caffeine application, the baseline frequency of sEPSPs was similar in both groups (two-way repeated-measures ANOVA; *F*_(1,51)_ = 0.295; *p* = 0.837; Figure 5A) and the frequency of sEPSPs was similarly increased by caffeine (paired *t*-test; *t* = −4.52; *p* < 0.001; Figure 5A). Prior to RyR activation, the baseline amplitude of sEPSPs was also similar in both the sham and rTBI groups (*F*_(1,51)_ = 0.98; *p* = 0.47; Figure 5B) and the amplitude of sEPSPs was similarly increased by caffeine (paired *t*-test; *t* = −8.14; *p* < 0.05; Figure 5B).

### Increased Phosphorylated Tau Staining After TBI Is Sustained Throughout the Cortex and Hippocampus

**Figure 6:**
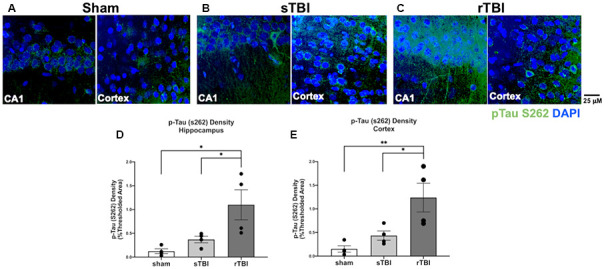
Hippocampal and cortical phosphorylated tau is elevated after single (sTBI) or repeated TBI (rTBI). **(A–C)** Representative confocal images (63×, multi-plane) show immunolabeling of phosphorylated tau (pTau S262, green) within the CA1 region of the hippocampus (left) and Layer V of the cortex (right) ipsilateral to injury in sham **(A)**, sTBI **(B)**, and rTBI **(C)** animals. **(D)** Bar graphs show a significant increase in hippocampal phosphorylated tau burden in the rTBI group (black) relative to the sTBI (gray) and sham groups (white; *n* = 16 slices/4 rats for each treatment condition). **(E)** Bar graphs show a significant increase in cortical phosphorylated tau burden in the rTBI group (black) relative to the sTBI (gray) and sham groups (white; *n* = 16 slices/4 rats for each treatment condition). Representative images were obtained on a Leica Microsystems SP8 confocal microscope. The injury was over the right sensorimotor cortex. *p ≤ 0.05 and **p ≤ 0.01. Error bars represent the standard error of the mean.

The level of phosphorylated tau protein burden was examined in the hippocampus and overlying cortex in the sham, sTBI, and rTBI treatment groups, ipsilateral and contralateral to the injury site, using immunohistochemical labeling and imaged using confocal microscopy (Figure 6). The density of each fluorophore above the background threshold level was averaged across all images obtained for a given animal using Metamorph software (v.7). In the hippocampus, this analysis revealed increased phosphorylated tau staining in the rTBI group compared to the sham group (one-way ANOVA; *F*_(2,11)_ = 7.242; *p* ≤ 0.01) and the sTBI group (*p* ≤ 0.05). In the overlying sensorimotor cortex, significant increases in phosphorylated tau in the rTBI group compared to the sham group (one-way ANOVA; *F*_(2,11)_ = 8.245; *p* ≤ 0.01) and the sTBI group (*p* ≤ 0.05) were also observed. There were no significant differences in phosphorylated tau between the ipsilateral and contralateral hemispheres. This pattern suggests an active process that is generating phosphorylated tau long after the initial injury occurred, though other explanations are possible such as impairments in the clearance of pathogenic protein species. Furthermore, the tau pathology is not necessarily limited to the site or hemisphere of injury, but distributes across brain hemispheres over time regardless of the initial TBI site, with a higher phosphorylated tau burden associated with a repetitive injury.

### Data Summary

**Figure 7:**
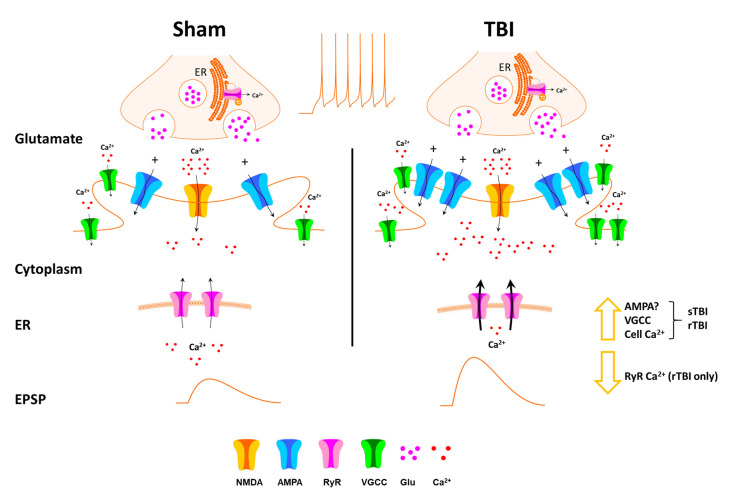
Schematic of effects of TBI on hippocampal synaptic transmission and Ca^2+^ handling. Thirty days after single (sTBI) or repeated TBI (rTBI), we observed potentiation of evoked fEPSPs, indicating synaptic potentiation, possibly resulting from postsynaptic effects including increased AMPA receptor effects. VGCC function was also increased in the sTBI and rTBI groups, and this, combined with increased synaptic transmission, may play a role in the increased basal cytosolic Ca^2+^ observed in both TBI groups. RyR-mediated Ca^2+^ release was decreased, but only in the rTBI group, possibly due to depletion of endoplasmic reticulum (ER) Ca^2+^ stores.

Overall, our study reveals a cluster of resilient pathogenic or maladaptive features of mild TBI that persist long after the acute effects of the initial injury. These features may collectively impact memory encoding systems in the hippocampus and may contribute to the dementia symptoms that can emerge long after the injury. In particular, we show that sTBI and rTBI result in increased excitatory synaptic output, along with increased resting Ca^2+^ and VGCC activity, and reduced RyR function in the rTBI group. This combination of increased synaptic excitability and increased VGCC activity may play a role in the elevated basal Ca^2+^ observed (for a graphical summary see Figure 7). We also observed increased and graded levels of phosphorylated tau in the sTBI and rTBI groups, strengthening the idea of an association between increased neuronal excitation and tau pathology.

## Discussion

Mild TBI or concussion has received considerable attention in recent years, in part reflecting increased awareness of its pervasiveness in the general public and its role as a significant risk factor for AD-like dementia and other neurological diseases. Despite this, few studies have examined and compared the persistent physiological and synaptic consequences of single or repeated, closed-head concussive events. To address the gaps in our understanding between brain injury and delayed cognitive deficits, we used a closed-head concussion model of single and repeated TBI that generates memory and performance deficits without overt hippocampal cell loss of detectable cell damage (Jamnia et al., 2017). One of the major limitations of most TBI studies to date has been the focus on measures of tissue damage or histopathology in fixed brain slices, a preparation in which active changes in real-time synaptic function cannot be detected. Here, we first employed extracellular field potential and whole-cell patch-clamp electrophysiological recordings to identify cellular and synaptic changes which may otherwise go undetected in fixed tissue. At the local circuit level in the hippocampus, we found a persistent upregulation of excitatory synaptic transmission at CA3-CA1 synapses which was present in both the single and repeated TBI groups 30 days after injury. Interestingly, these fundamental changes in synaptic responses did not result in altered expression of long-term synaptic plasticity; however, this does not preclude underlying LTP deficits in this injury model as the recruitment of compensatory mechanisms to sustain normal-appearing LTP has been demonstrated in compromised hippocampal networks (Chakroborty et al., 2012). There also were no explicit alterations in paired-pulse facilitation, which is generally accepted as an index of evoked presynaptic release probability (Regehr, 2012). Interestingly, rTBI also had no effect on the frequency of mEPSCs measured using whole-cell recordings, reinforcing the observation that TBI does not affect the presynaptic release probability weeks after injury. Although this does not rule out a presynaptic component due to an acute but transient increase in synaptic glutamate release in the wake of TBI (Huang et al., 1992).

As our data indicate that hippocampal synaptic transmission is similarly increased by either sTBI or rTBI, this suggests that even a single concussive event is sufficient to elicit long-term synaptic effects. Although the exact series of cellular events which lead to this potentiated synaptic state is still under investigation, our data suggest that it is a postsynaptic component of the fEPSP that is potentiated. As the fEPSP is typically mediated by activation of postsynaptic AMPA receptors (Davies and Collingridge, 1989; Tsien et al., 1996), upregulation of AMPA receptors may play a role in the synaptic potentiation observed. Additionally, as the resting membrane potential of CA1 pyramidal neurons was approximately −70 mV in all treatment groups (Table 1), it is unlikely that there is a substantial NMDAR fEPSP component due to the Mg^2+^ block of NMDARs at this hyperpolarized membrane potential. Circuit hyper-responsivity may also reflect decreased RyR function and downstream Ca^2+^-regulated neuromodulators (Chakroborty et al., 2015), as well as pathological tau accumulation (Huijbers et al., 2019). Tau levels are associated with seizure incidence (DeVos et al., 2013), a well-documented consequence of TBI that may also occur as a result of hippocampal hyperactivity (Golarai et al., 2001). Another possible explanation for the hippocampal hyperexcitability observed is decreased CA1 hippocampal inhibitory neurotransmission, another feature shared in animal models of both TBI and Alzheimer’s disease (Reeves et al., 1997; Hazra et al., 2013; Almeida-Suhett et al., 2015).

**Table 1:** Passive and active membrane properties are unchanged by single or repeated traumatic brain injury (TBI).

	Sham	sTBI	rTBI
Cell Potential (mV)	−70.8 ± 0.35 (*n* = 18)	−70.4 ± 0.17 (*n* = 18)	−70.6 ± 0.19 (*n* = 24)
Input Resistance (MΩ)	101 ± 5.23 (*n* = 18)	112 ± 5.77 (*n* = 18)	118 ± 7.71 (*n* = 24)
Sag (%)	24.8 ± 1.14 (*n* = 18)	25.1 ± 1.03 (*n* = 18)	27.2 ± 0.98 (*n* = 24)
Spike Threshold (mV)	−50.0 ± 1.41 (*n* = 11)	−46.1 ± 0.82 (*n* = 16)	−48.1 ± 1.00 (*n* = 19)
Spike Amplitude (mV)	102 ± 2.14 (*n* = 11)	97.1 ± 2.1 (*n* = 16)	99.4 ± 1.83 (*n* = 19)
Spike Half-Width (ms)	1.82 ± 0.04 (*n* = 11)	1.98 ± 0.06 (*n* = 16)	1.89 ± 0.05 (*n* = 19)

*Passive and active membrane properties of hippocampal CA1 pyramidal neurons, including resting membrane potential, input resistance, and action potential characteristics were unchanged by either single TBI (sTBI) or repeated TBI (rTBI)*.

The decreased RyR-evoked Ca^2+^ release in the rTBI group is similar to the reported decreased RyR-Ca^2+^ response resulting from mechanical neuronal injury *in vitro* (Weber et al., 2002), an effect mediated by NMDAR mediated Ca^2+^ influx (Ahmed et al., 2002), subsequent increased CICR, and depletion of ER Ca^2+^ stores (Weber et al., 2002). Indeed, elevated basal cytosolic Ca^2+^ resulting from a single TBI event is prevented by pretreatment with the NMDAR antagonist MK801 (Deshpande et al., 2008). Although we did not observe decreased RyR-Ca^2+^ effects in the sTBI group, it is plausible that effects of repeated mechanical injury and repeated NMDAR Ca^2+^ influx are more persistent than those arising from a single injury (Slemmer et al., 2002; Weber, 2007), resulting in long-term modification of RyR-Ca^2+^ signaling as a result of rTBI. Notably, the sustained rTBI effect on RyR-Ca^2+^ correlates with persistent cognitive impairments resulting from rTBI, but not sTBI (Jamnia et al., 2017), further strengthening the association between RyR dysfunction, Ca^2+^ dyshomeostasis, and cognitive impairment after brain injury (Deshpande et al., 2008; Sun et al., 2008; Bruno et al., 2012; Liu et al., 2012).

While decreased RyR-Ca^2+^ was observed in the rTBI group only, increased VGCC activity was observed in both treatment groups, and may play a role in the elevated basal Ca^2+^ present in both TBI groups. The sustained upregulation of hippocampal synaptic transmission observed in both TBI groups may also be driven by the increased Ca^2+^ influx through VGCCs, and there is ample evidence that VGCCs play an important role in hippocampal synaptic potentiation (Huang and Malenka, 1993; Huber et al., 1995). As there was no significant effect of either sTBI or rTBI on the passive or active cell membrane properties measured 30 days post-injury (Table 1), the increased VGCC responses observed cannot be explained by changes in membrane resistance or postsynaptic action potential threshold.

Tau pathology is a well-documented feature of TBI and related conditions such as chronic traumatic encephalopathy (CTE) and Alzheimer’s disease (McKee et al., 2013; McKee and Robinson, 2014; Albayram et al., 2016), yet the defined mechanisms by which tau phosphorylation is sustained after an injury have yet to be fully elucidated. Across TBI conditions, the uniformly distributed pathogenic tau seen 30 days post-injury is likely resulting from a feed-forward cascade, as the half-life of soluble tau is approximately 10 days (Yamada et al., 2015). A possible mechanism is the increased resting cytosolic Ca^2+^ levels following TBI, which can upregulate GSK3β and Cdk5 activity and phosphorylate tau at serine and threonine residues (Avila et al., 2004). This in turn can disrupt Ca^2+^ homeostasis, and sustain the pathogenic cycle (Gómez-Ramos et al., 2006). Consistent with this, GSK3β inhibitors have shown therapeutic potential for treating TBI beyond a reduction in phosphorylated tau, presumably by altering signaling *via* receptor tyrosine kinases (RTKs) and Wnt pathways, effects that would otherwise lead to the accumulation of pathological proteins, neuronal loss, and cognitive dysfunction (Shim and Stutzmann, 2016). In addition to altering Ca^2+^ homeostasis, abnormally phosphorylated tau disrupts microtubule assembly and axonal transport, leading to synaptic deficits and ultimately synaptic loss (Ial et al., 2005). Phosphorylated tau may also rapidly propagate and accumulate in brain regions distant from, but synaptically connected to, the site of injury following TBI (Edwards et al., 2020). Additional mechanisms of widespread histopathology may include contrecoup injury or generalized neuroinflammation (Edwards et al., 2020). The exact processes accounting for the propagation and distribution of tau pathology at this 30-day time point require further study.

Despite this evidence demonstrating alterations in synaptic physiology after TBI, much remains to be determined regarding the nature and source of sustained physiological changes in a closed-head injury model and how this relates to plasticity and memory deficits long after the initial components of the injury have healed. Ca^2+^ dyshomeostasis is a central pathogenic element and has been observed in TBI models at several time points (Deshpande et al., 2008; Sun et al., 2008; Weber, 2012). Ca^2+^ signaling is centrally involved in synaptic transmission, plasticity, and tau phosphorylation, and thus can generate a self-sustaining pathogenic cycle, as seen in chronic neurodegenerative disorders such as Alzheimer’s disease (Stutzmann, 2007; Chakroborty et al., 2009, 2012, 2019). In the rTBI group, the blunted RyR-Ca^2+^ response may reflect reduced ER Ca^2+^ stores resulting from leaky or sensitized RyRs; this leak may also contribute to the elevated resting cytosolic Ca^2+^ levels observed (Lacampagne et al., 2017). Furthermore, increased Ca^2+^ entry through VGCCs, coupled with increased synaptic excitation may also contribute to the elevated Ca^2+^ load. Elevated cell Ca^2+^ can subsequently increase PKA activity, which phosphorylates and subsequently sensitizes the RyR to leak excess ER Ca^2+^ into the cytosol (Morimoto et al., 2009; Liu et al., 2012; Lacampagne et al., 2017), thus lowering ER Ca^2+^ reserves and reducing evoked responses while contributing to increased cytosolic resting levels, as is observed in this study.

Several clinical studies have demonstrated positive effects of FDA-approved Ca^2+^ channel blockers such as nimodipine on improved cognitive function in TBI patients (Teasdale et al., 1990, 1992; Bailey et al., 1991), however, subsequent analyses have reported no effects on mortality rates (Langham et al., 2003; Vergouwen et al., 2007). Likewise, the RyR negative allosteric modulator dantrolene attenuates glutamate excitotoxicity *in vitro* (Frandsen and Schousboe, 1991) and *in vivo* (Niebauer and Gruenthal, 1999), and normalizes intracellular Ca^2+^ homeostasis in AD models (Chakroborty et al., 2012, 2019), thus raising the possibility that altered VGCC and RyR mediated Ca^2+^ signaling play a central role in the long term consequences of TBI. Given that VGCCs and RyRs may work in a feed-forward manner to sustain TBI pathophysiology, combined targeting of these channels may serve as an effective therapeutic strategy in the treatment of TBI.

Although our study extends 30 days after TBI, further investigation is warranted to determine whether these effects persist for longer periods, especially as hippocampal hyperactivity may precede synaptic depression and neurodegeneration (Huijbers et al., 2015). Also, human studies indicate cognitive effects lasting months to years (McInnes et al., 2019; Shen et al., 2020), indicating the need for animal studies of analogous duration.

## Conclusions

Here, we demonstrated that both single and repeated mild TBI result in a similarly persistent upregulation of hippocampal synaptic transmission, effects accompanied by altered Ca^2+^ homeostasis and phosphorylated tau expression. These data strengthen the idea that a single concussive event can result in persistent brain pathophysiological effects that are similar in magnitude to those observed after repeated concussions, with future implications for behavior, learning, and memory.

## Data Availability Statement

The raw data supporting the conclusions of this article will be made available by the authors, without undue reservation.

## Ethics Statement

The animal study was reviewed and approved by Rosalind Franklin University IACUC DePaul University IACUC.

## Author Contributions

JM, CB, and NB conducted the experiments and analyzed data, and contributed to the writing of manuscript. DP, DK, and GS contributed to the experimental design, oversight, execution of the study, and writing of the manuscript. All authors contributed to the article and approved the submitted version.

## Conflict of Interest

The authors declare that the research was conducted in the absence of any commercial or financial relationships that could be construed as a potential conflict of interest.
